# Recruiting controls from an online panel for a case–control study enabled a timely and reliable foodborne *Salmonella* outbreak investigation, Germany 2021

**DOI:** 10.1017/S0950268823000493

**Published:** 2023-04-05

**Authors:** Anna Łuczyńska, Johannes Dreesman, Elke Mertens, Mareike Wollenweber, Delphine Perriat, Bettina M Rosner

**Affiliations:** 1 ECDC Fellowship Programme, Field Epidemiology Path (EPIET), European Centre for Disease Prevention and Control, Stockholm, Sweden; 2 Department for Infectious Disease Epidemiology, Public Health Agency of Lower Saxony, Hanover, Germany; 3 Department of Infectious Disease Epidemiology, Robert Koch Institute, Berlin, Germany

**Keywords:** Case–control study, infectious disease, online panel, outbreaks, *Salmonella* Braenderup

## Abstract

We explored the feasibility, suitability, and reliability of using controls recruited among members of a non-probabilistic online panel (‘panel controls’) in a case–control study (CCS) to investigate a *Salmonella* Braenderup outbreak in Germany. For comparison, another control group was recruited via random digit dialling (‘classical controls’). Panel members received questionnaires by email; classical controls were interviewed by phone. Both control groups were frequency-matched to cases by age and sex; the classical controls also by federal state. Cases and controls were queried mainly about fruit consumption since melons were the suspected infection vehicle. We calculated adjusted odds ratios (aOR) and 95% confidence intervals (CIs) using single-variable and multivariable logistic regression. The study included 32 cases, 81 panel controls and 110 classical controls. Analyses identified melons, particularly Galia melons, as the most likely infection vehicle using either control group (panel controls – aOR 12, CI 2.7–66; classical controls – aOR 55, CI 8–1100). Recruitment of panel versus classical controls required substantially less person-time (8 vs. 111 hours) and was about 10 times less expensive. We recommend this timely and reliable control recruitment method when investigating diffuse foodborne outbreaks with CCS.

## Introduction

Foodborne diseases are a significant public health concern in Europe and worldwide [1, 2]. In Germany, an average of approximately 330 foodborne outbreaks with 1100–1400 related cases per year have been reported from 2015 to 2019, according to the Protection against Infection Act (*Infektionsschutzgesetz*; IfSG). In less than half of the reported outbreaks (about 45%), one or more food items were named as suspected infection vehicles, mostly with weak evidence only. Specific food items that cause outbreaks often remain unknown [3, 4]. The effective control of foodborne outbreaks relies on the timely identification of suspected infection vehicles along with the rapid implementation of suitable control measures [5].

As a method for hypotheses testing regarding the likely source of an outbreak, a case–control study (CCS) can be a key component of a foodborne outbreak investigation [6]. In a typical CCS, the recruitment of cases is often straightforward because cases are notified to the local health authorities, who in turn will help contact and recruit cases. Classical methods of control recruitment during foodborne outbreaks include case- or physician-nominated controls, nearest-neighbour recruitment, population registers, or random digit dialling (RDD) [5, 7, 8]. Not only can these recruitment strategies be time- and resource-consuming, but also controls are usually difficult to reach (e.g. not responding to unsolicited calls, not available at daytime), or not willing to participate in studies. Many persons need to be contacted before sufficient number of controls can be sampled, and low response rates lead to concerns regarding selection bias [9, 10]. Therefore, CCS is rarely employed, even if indicated [9, 11].

Pre-established panels of consenting participants have recently been used to recruit controls for CCS and allowed efficient investigations of foodborne outbreaks as they required less human, time, and financial resources compared to classical control recruitment methods [9, 12, 13]. Further investigation is now required to understand potential biases that may be introduced by this method. The HuGO online panel (*Hygiene und Gesundheit Online-Befragung* (Hygiene and Health Online Survey)) was established in 2018 by the Public Health Agency of the federal state of Lower Saxony, Germany (NLGA). Currently 271 participants, who volunteered to answer health and hygiene-related online questionnaires regularly, are enrolled [14]. In a previous study, which used data from four completed outbreak investigations, it was retrospectively shown that the use of the panel as a control group in CCS allowed a successful identification of infection vehicles [14].

The present study intended to use the HuGO panel as a control group in a CCS in an ongoing foodborne outbreak investigation in parallel with the recruitment of controls via RDD – an accessible and appropriate method to recruit controls for CCS in an outbreak context.

Between 15 March and 6 July 2021, 348 laboratory-confirmed *Salmonella (S.)* Braenderup cases were reported in 12 different EU/EEA countries and the UK. Based on epidemiological and trace-back investigations in other countries, the vehicles of infection were presumed to be melons imported from South or Central America [15]. More than 80 *S.* Braenderup outbreak cases were identified in Germany.

This study aimed to explore the feasibility, suitability, and reliability of using the HuGO panel as a control group in a CCS to investigate the *S.* Braenderup outbreak in Germany in 2021.

## Methods

### Recruitment and interviewing of cases

Cases were recruited from all *S.* Braenderup outbreak cases (probable or confirmed cases) that were identified in Germany and notified to the local health authorities, according to the IfSG.

*S.* Braenderup cases were defined as:

- *confirmed* outbreak cases if
  - a corresponding *S.* Braenderup sequence type (ST) 22 isolate was received at the national reference centre (NRC) or at the Institute of Hygiene and Environment (HU Hamburg), in weeks 11–30 (March–July 2021) and
  - the *S.* Braenderup isolate belonged to the core genome multilocus sequence typing (cgMLST) – cluster ‘SAL_Braenderup_2021_NGS_1’ – within five allelic differences.
- *probable* outbreak cases if
  - the reported date of disease onset was within weeks 11–30 (or notification date, if disease onset date was missing) and
  - cgMLST information from a corresponding isolate was not obtained at the NRC or HU Hamburg.

Confirmed and probable outbreak cases were contacted by local health authorities and requested to participate in the study. Only persons who gave oral informed consent were included as study participants.

Cases were interviewed by phone between 26 May and 23 July 2021 by the employees either of the German National Public Health Institute (Robert Koch Institute, RKI) or of the respective federal state health authorities in the states of their residence. If cases were minors, their parents were interviewed on their behalf.

### Recruitment of classical controls

A ‘classical control’ was defined as a person who met the case-matching criteria regarding age, sex, and federal state of residency and gave verbal consent to participate in the study. Adult controls were frequency-matched to adult cases based on age group (18–35 years; 36–59 years; 60–79 years; 80 years and older), sex, and federal state of residency. Children controls were frequency-matched to children cases based on age group (1–5 years; 6–14 years) and federal state of residency (one of any of the federal states with children cases of the same age group). No controls were recruited in the age group 15–17 years, because there were no cases in this age group. Controls were recruited via RDD in the period between 1 and 27 July 2021 and interviewed by phone by the employees of a market and social research institute on behalf of the RKI. Interviews with potential controls were aborted if they reported diarrhoea within 7 days prior to the interview (exclusion criterion). The ratio of cases to controls was 1:3.

### Recruitment of panel controls

‘Panel controls’ were recruited from the HuGO panel. The panel consists of 271 adults living in Lower Saxony, Germany, who volunteered to regularly respond to online surveys on health-related topics. Details on the panel are available elsewhere [14]. On 1 July 2021, all panel members were invited via e-mail to participate in the study and complete a web-based questionnaire created with the LamaPoll software (https://www.lamapoll.de/Umfrage-Software). Participants who declared that they had children aged 1–14 years were requested to fill in the questionnaire for the one child whose birthday (day/month) was most recent. Controls were excluded from data analysis if they reported diarrhoea within 7 days prior to answering the questionnaire, or if they answered the questionnaire later than 27 July 2021. Before analysis, the panel controls were frequency-matched to cases on sex and age group.

### Data protection and consent to participate

The study adhered to the EU’s general data protection regulation. Participation in the study was voluntary. Informed consent was obtained from all persons participating in the study, or from a parent or legal guardian in case of minors. Participants who were interviewed by phone (cases and classical controls) gave verbal consent before the interview started. Panel controls gave informed consent in an online form before answering the web-based questionnaire.

### Data collection

Data was collected using a standardised questionnaire. This questionnaire was based in the UK in a CCS to investigate the multinational *S.* Braenderup outbreak.

For all study participants, sociodemographic data (e.g. age, sex, level of education) as well as data on general food consumption habits (e.g. vegetarian or other diet) were collected. Cases were questioned about their salmonellosis (e.g. date of onset, duration, hospitalisation, possible transmission in the household). They were also asked if their work involved handling food, and if they ate certain fruit items within the 7-day period before disease onset.

For classical and panel controls, data on the consumption of 27 fresh fruit items in the 7 days before the interview was collected. In addition, the consumption of a subset of 10 fruit items in the 7 days following Easter Sunday 2021 (5–11 April 2021) was queried. The subset included nine fruit items, whose consumption was assumed to vary substantially according to the season (various types of melons: Galia, cantaloupe, honeydew and others, and watermelon), berries (strawberries, raspberries, blueberries, blackberries), and one fruit item (apples) for which a seasonal variation in consumption was assumed to be less likely. The 7-day time period following Easter Sunday was chosen because it was a part of the time period of disease onset among cases (31 March–24 May 2021). Additionally, we assumed that food consumption would be easier to remember if queried in relation to a landmark in time, such as Easter [16].

### Data analysis

The feasibility of using the panel as a source for controls was assessed by comparing the human, time, and financial resources that were required to recruit panel controls, with those required to recruit classical controls by a market and social research institute. To calculate the response rate for panel controls, the number of panel members who answered the questionnaire and met eligibility criteria was divided by the total number of panel members contacted by e-mail. To calculate the response rate for classical controls, the number of successful interviews was divided by the number of contacted households with persons eligible for interviewing.

The suitability of panel controls was analysed by comparing their sociodemographic characteristics (age, sex, education) to those of classical controls, using chi-squared tests, and by comparing both control groups regarding food exposures using Fisher’s exact tests.

The reliability of the panel as a source for controls was assessed by comparing the analytical findings between the two control groups deployed in parallel in the CCS. We explored associations between the consumption of a variety of food items and the disease. First, unconditional logistic regressions based on single-exposure variables adjusted for age group and sex (‘single-variable analyses’) were performed to estimate adjusted odds ratios (aORs) with 95% confidence intervals (CIs).

Variables which were positively associated with the disease in single-variable analyses with *p*-values <0.1 were included in multivariable models, together with age group and sex. Variables were then removed using stepwise backward elimination. Variables with *p*-values <0.05 and the variables sex and age group remained in the final multivariable model. We created four models. In models A and C, we considered data on the frequency of food consumption by controls in the 7 days before the interview, that is, in July 2021, whereas in models B and D, we used data on the frequency of food consumption in the 7 days after Easter Sunday, that is, in April 2021. Additionally, models C and D included variables for individual types of melons, whereas in models A and B, a composite ‘any melon’ variable comprising Galia, cantaloupe, honeydew, or similar types of melon, but not watermelon, was included.

The four multivariable models using panel controls were then compared to the four corresponding models using classical controls. The results were considered similar between the corresponding models if exposures were significantly positively associated (*p*-values <0.05; aOR >1) or significantly negatively associated (*p*-values <0.05; aOR <1) with being a case in the model using panel controls and in the corresponding model using classical controls.

All analyses were carried out using R statistical software (version 4.0.2) or Stata (version 17.0).

## Results

Of the 84 outbreak cases, 35 (42%) were interviewed. Three cases were excluded from the study because the date of disease onset remained unclear (*n* = 1), the person was a child younger than 1 year (*n* = 1) or the person was interviewed after the recruitment of the control groups ended (*n* = 1). The remaining 32 cases (23 adults, 9 children) were included in the study (38% of all outbreak cases). A total of 110 classical controls (83 adults, 27 children) were interviewed, and 164 panel members completed the online questionnaire (134 for adults and 30 for children). Datasets from panel members who reported diarrhoea within 7 days before the interview (*n* = 5), or provided incomplete data (*n* = 1), or answered after 27 July 2021 were excluded from analyses. From the remaining 156 panel controls, 81 were randomly selected to frequency-match cases in terms of sex and age group (56 adults and 25 children) (Supplementary Table S1).

### Feasibility

#### Human and time resources

Finding, recruiting, and interviewing classical controls by telephone required 129 hours in total: 18 hours was the actual interview time (about 10 minutes per interview) and 111 hours were required for finding and recruiting the 110 interviewees, taking case-matching criteria into account. Inviting panel members to participate in an online survey was performed by one scientist at the NLGA. It took 8 hours to integrate the questionnaire into the survey software and distribute the survey link to panel controls via e-mail (Table 1).

**Table 1.** Comparison of recruitment methods and feasibility parameters between classical and panel controls: case–control study to investigate the *S.* Braenderup outbreak, Germany, 2021

	Classical controls	Panel controls
Control recruitment and data collection			
Control selection	Random digit dialling taking matching criteria into account	Selection of panel members according to matching criteria
Data collection	Computer-assisted telephone interviewing	Online survey distributed via e-mail Reminder sent after 1 week
Matching criteria	*Adult controls* frequency-matched on age group (18–35; 36–59; 60–79, ≥80 years), sex, federal state of residency *Children controls* frequency-matched on age group (1–5; 6–14 years) and federal state of residency Intended case–control ratio 1:3	*Adult controls* frequency-matched on age group (18–35; 36–59; 60–79, ≥80 years) and sex *Children controls* frequency-matched on age group (1–14 years) and sex Intended case–control ratio 1:3
Number of controls with complete datasets	110	Before frequency matching: 156 After frequency matching: 81
Detailed activities^a^	Programming questionnaire (VOXCO Command Center) Training interviewers Calling phone numbers and finding controls according to inclusion criteria Interviewing suitable controls and collecting data via CATI	Creating online questionnaire with Lamapoll software Sending e-mails to panel members Managing database, including applying exclusion criteria
Financial resources			
Total price	3000–5000€	290€
Service provider	Private market and social research institute	Public health agency of Lower Saxony
Human and time resources			
Person-time	129 hours	8 hours
Person-time per control	About 1.2 hours	About 6 minutes
*Response rate*	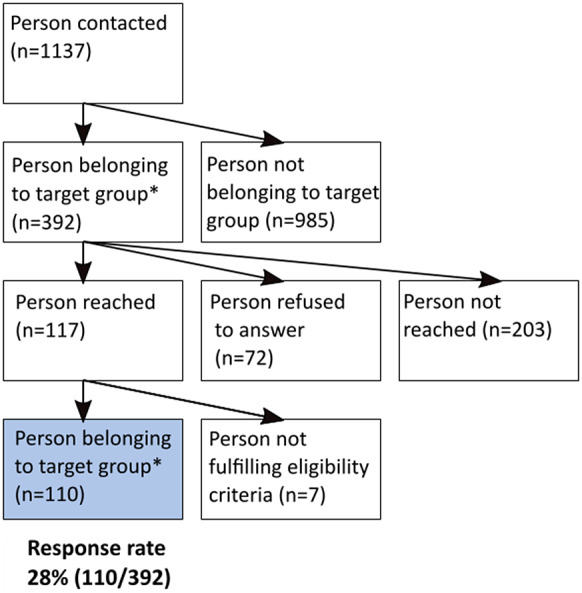	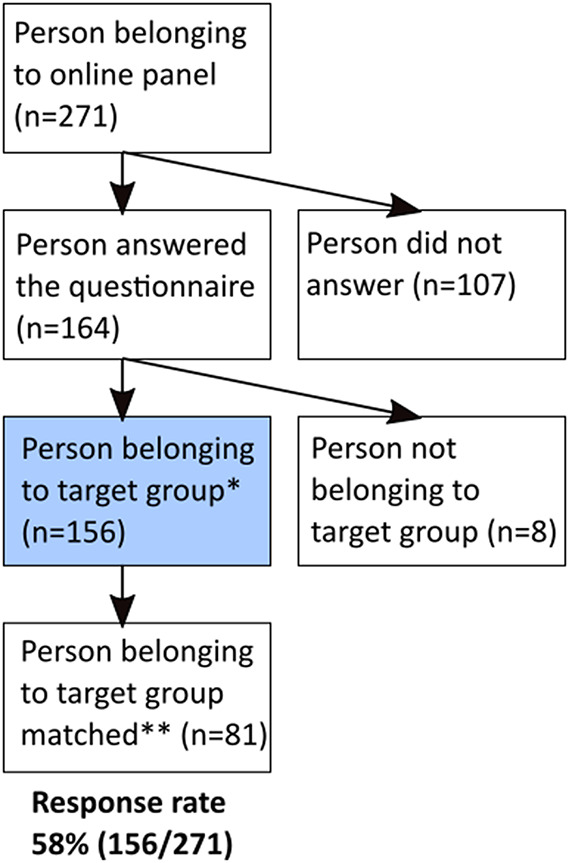
	*Target group: persons who fulfilled matching criteria (i.e. age group, sex, federal state for adults; or age group and federal state for children) and eligibility criteria	*Target group: persons who fulfilled eligibility criteria **Frequency matching criteria: age group and sex

a The time required to develop the questionnaire, introductory text, and other organisational input was considered equal for both sets of controls.

#### Financial resources

The total costs for recruiting and interviewing classical controls were 3000–5000€, including value-added tax (exact costs are considered a business secret and cannot be disclosed). Expenses for recruiting panel controls comprised costs for survey software and a scientist’s salary for 8 hours, 290€ in total (Table 1).

#### Response rate

For the recruitment of classical controls, 1377 households were contacted. Of 392 eligible persons, 110 met the case-matching criteria and could be interviewed successfully, resulting in a response rate of 28%. Of the 271 panel members who were invited to answer the online questionnaire, 156 answered within the study period, resulting in a response rate of 58%.

### Suitability

Before being frequency-matched to cases on sex and age group, panel controls had a similar sex distribution compared to classical controls (Table 2; 59% vs. 64% female; *p* = 0.42). Panel and classical controls differed significantly with respect to age (panel controls were older than classical controls; however, classical controls had to fulfil case-matching criteria before recruitment) and level of education (panel controls had a higher education level). The age difference between panel and classical controls became insignificant after frequency-matching panel controls to cases.

**Table 2.** Distribution of sociodemographic characteristics among cases, frequency-matched classical controls, panel controls, and frequency-matched panel controls: case–control study to investigate the *S.* Braenderup outbreak, Germany 2021

	Cases, *n* (%)	FM classical controls^a^ *n* (%)	Panel controls *n* (%)	FM panel controls^b^ *n* (%)		
	(*n*_tot_ = 32)	(*n*_tot_ = 110)	(*n*_tot_ = 156)	(*n*_tot_ = 81)	*p*-value (chi^2^) FM classical controls vs. panel controls	*p*-value (chi^2^) FM classical controls vs. FM panel controls
Gender					0.42	0.34
Male	13 (41)	40 (36)	64 (41)	35 (43)		
Female	19 (59)	70 (64)	91 (59)	46 (57)		
Age (years)					<0.001	0.54
1–17	9 (28)	27 (25)	25 (16)	25 (31)		
18–35	9 (28)	27 (25)	14 (9)	14 (17)		
36–59	4 (12)	19 (17)	61 (39)	12 (15)		
≥60	10 (31)	37 (34)	56 (36)	30 (37)		
Education level^c^					0.003	0.027
No postsecondary education	NA	13 (12)	1 (1)	1 (1)		
Apprenticeship	NA	34 (31)	51 (35)	20 (26)		
Technical college	NA	13 (12)	16 (11)	7 (9)		
University of applied science	NA	13 (12)	22 (15)	10 (13)		
University	NA	35 (32)	56 (38)	38 (50)		

a FM classical controls: frequency-matched classical controls, that is, adults recruited through a classical method (random digit dialling) who are frequency-matched to adult cases on age group, sex, and federal state of residency; or children recruited through a classical method (random digit dialling) who are frequency-matched to children cases by age group and federal state of residency.

b FM panel controls: frequency-matched panel controls, that is, adult panel members who are frequency-matched to adult cases on sex and age group; or children of adult panel members who are frequency-matched to children cases on sex and age group.

c German translations: no postsecondary education: *kein Berufsabschluss*; apprenticeship: *Lehre/Berufsausbildung*; technical college: *Fachschulabschluss*, university of applied science: *Fachhochschule/Berufsakademie*; university: *Universität/Promotion.*

NA, not available; *n*_tot_, total number of participants in the study group.

Frequency-matched panel controls reported a similar frequency of melon consumption than classical controls in the 7 days after Easter Sunday (8.6% vs. 5.5%; aOR 1.6, 95% CI 0.45–6.2) (Table 3). They also reported a similar frequency of consumption of the 21 other fruit items that were queried, except for cherries and mangoes (Supplementary Tables S2 and S3).

**Table 3.** Comparison of exposures to melons, watermelon, and special eating habits between classical controls (reference) and panel controls during the 7-day time period after Easter Sunday (05–11 April 2021): case–control study to investigate the *S.* Braenderup outbreak, Germany, 2021

	Exposed FM classical controls^a^ *n* (%)	Exposed FM panel controls^b^ *n* (%)	Exposed FM classical controls vs. exposed FM panel controls	
	(*n*_tot_ = 110)	(*n*_tot_ = 81)	aOR [95% CI]	*p*-value (Fisher’s exact test)
Melons				
Any melon^c^	6 (5.5)	7 (8.6)	1.6 [0.45–6.2]	0.40
Galia melon	1 (0.91)	3 (3.7)	4.2 [0.3–222]	0.31
Cantaloupe melon	2 (1.8)	1 (1.2)	0.68 [0.01–13]	1.00
Honeydew melon	6 (5.5)	5 (6.2)	1.1 [0.26–4.7]	1.00
Watermelon	13 (12)	9 (11)	0.93 [0.33–2.5]	1.00
Eating habits				
Any special diet	17 (15)	13 (16)	1.08 [0.45–2.5]	0.84
Vegetarian	7 (6.4)	9 (11)	1.9 [0.59–6.3]	0.29
Vegan	2 (1.8)	1 (1.3)	0.69 [0.01–14]	1.0

a FM classical controls: frequency-matched classical controls, that is, adults recruited through a classical method (random digit dialling), who are frequency-matched to adult cases on age group, sex, and federal state of residency; or children recruited through a classical method (random digit dialling), who are frequency-matched to children cases by age group and federal state of residency.

b FM panel controls: frequency-matched panel controls, that is, adult panel members who are frequency-matched to adult cases on sex and age group; or children of adult panel.

c Galia or cantaloupe or honeydew melon or similar types of melon, but not watermelon (composite variable).

aOR, adjusted odds ratio; CI, confidence interval; *n*_tot_, total number of participants in the study group; missing values: eating habits (*n* = 2).

### Reliability

#### Single-variable analyses

When the 7-day exposure time period after Easter Sunday, in April, was used for controls, cases were more likely to have eaten melons than classical controls (aOR 32 for the variable ‘any melon’; CI 11–120) (Supplementary Table S4). They were also more likely to have eaten specific melon types than classical controls: Galia (aOR 96, CI 15–2100), cantaloupe (aOR 29; CI 6.2–230), and honeydew (aOR 10; CI 3.6–34). Similarly, cases were more likely to have eaten melons than panel controls (aOR 19; CI 6.3–71), and also more likely to have eaten specific melon types: cantaloupe (aOR 27; CI 4.4–540), Galia (aOR 18; CI 4.6–100), and honeydew (aOR 8.6; CI 2.7–31). Details of the single-variable analyses with all food items are provided in Supplementary Table S4.

Although aORs were lower, similar associations could be shown for melon consumption during the 7 days before the interview, in July (Supplementary Table S5).

#### Multivariable analyses

Table 4 presents the results of the multivariable analyses of the CCS using classical or panel controls. Using either control group, melon consumption (variable ‘any melon’) was found to be strongly and statistically significantly associated with the disease, confirming the results of the single-variable analyses (models A and B). For the 7-day exposure period in July, melon consumption was significantly associated using either control group (model A, Table 4). This association was even stronger when the 7-day period after Easter Sunday (in April) was used as exposure time period for controls (model B).

**Table 4.** Final models of the multivariable logistic regression analyses in which the fruit consumption of cases was compared to the fruit consumption of frequency-matched classical controls and frequency-matched panel controls: case–control study to investigate the *S.* Braenderup outbreak, Germany, 2021

	Model characteristics			Exposed FM classical controls^a^ (*n*_tot_ = 110)			Exposed FM panel controls^b^ (*n*_tot_ = 81)		
Model	Exposure time period for controls	Variable related to melon exposure	Variables included in the final model	aOR	95% CI	*p*-value	aOR	95% CI	*p*-value
A	7 days before the interview (July 2021)	Any melon^c^	Any melon	7.2	2.5–20	<0.001	11	3.6–40	<0.001
			Apples	8.0	1.5–44	0.017	n.s.	n.s.	n.s.
			Oranges	6.5	1.9–22	0.003	4.5	1.3–16	0.017
			Kiwi	n.s.	n.s.	n.s.	5.4	1.5–21	0.01
B	7 days after Easter Sunday (April 2021)	Any melon^c^	Any melon	32	11–121	<0.001	19	6.3–71	<0.001
C	7 days before the interview (July 2021)	Individual melon types	Galia melon	32	7.3–190	0.001	6.2	1.4–31	0.020
			Cantaloupe melon	n.s.	n.s.	n.s.	6.6	1.1–41	0.037
			Apples	10	2.02–98	0.015	n.s.	n.s.	n.s.
			Oranges	9.7	2.9–36	<0.001	5.1	1.5–19	0.012
			Kiwi	n.s.	n.s.	n.s.	5.6	1.5–23	0.011
D	7 days after Easter Sunday (April 2021)	Individual melon types	Honeydew melon	4.7	1.2–18	0.024	5.1	1.3–21	0.017
			Galia melon	55	8.2–1100	0.001	12	2.7–66	0.002

a FM classical controls: frequency-matched classical controls, that is, adults recruited through a classical method (random digit dialling), who are frequency-matched to adult cases on age group, sex, and federal state of residency; or children recruited through a classical method (random digit dialling), who are frequency-matched to children cases by age group and federal state of residency.

b FM panel controls: frequency-matched panel controls, that is, adult panel members who are frequency-matched to adult cases on sex and age group; or children of adult panel.

c Galia or cantaloupe or honeydew melon or similar types of melon (composite variable).

aOR, odds ratio adjusted for age and sex; CI, confidence interval; n.s., not significant (not included in the final model).

When differentiating various melon types, the consumption of Galia melons (models C and D) had the strongest association with the disease, when using both control groups and both exposure time periods for controls. For example, cases were 55 times more likely to have eaten Galia melons than classical controls and 12 times more likely than panel controls when the 7-day time period after Easter Sunday was used as exposure time period for controls (model D).

In the final models A and C, the consumption of some other fruits was statistically significantly associated with the disease. These were oranges and apples, when cases were compared to classical controls; and oranges and kiwi, when cases were compared to panel controls (Table 4). However, those associations were weaker than those for the consumption of ‘any melon’ and Galia melons.

## Discussion

This study was performed in the context of a multinational *S.* Braenderup outbreak, which affected Germany as well as several other European countries from March to July 2021 [15]. The investigation identified melons, in particular Galia melons, as the infection vehicle [17].

The three most affected countries in the outbreak – UK, Denmark (DK), and Germany – performed country-specific CCS in May (UK and DK) and July 2021 (Germany). The UK CCS confirmed that Galia melons were the most likely infection vehicle, whereas the DK CCS remained inconclusive [17].

Recruiting and interviewing controls using an online panel for the CCS was feasible and cost-efficient compared to recruiting and interviewing classical controls. Moreover, results of the CCS using the online panel were quickly obtained and allowed an earlier confirmation of Galia melons as the most likely infection vehicle. These findings are in line with several case–control studies that employed commercial online panels as a source of controls to investigate foodborne outbreaks and demonstrated time and cost savings in comparison to the recruitment of controls among public health staff members [12], or other approaches such as random or sequential digit dialling, and were not influenced by bias in regard to source or vehicle of infection [9]. A previous study comparing the use of existing databases in the possession of major retailers (control banks) to RDD proved to be more time- and resource-efficient [13].

Members of an online panel were shown to be suitable as controls in this CCS, as they did not substantially differ from classical controls in terms of age group and sex, after being frequency-matched to cases [5]. However, panel members had a higher education level than classical controls. A possible explanation may be that persons with a higher education level are more likely to participate in an online panel to support public health topics [18]. This selection bias is not unique to online panels and has been observed with other control recruitment methods for CSS, including RDD [19]. Currently, there is no single standard method for selecting controls [5] as all available recruitment methods are subjected to limitations and inherent biases [12]. We demonstrated that the use of online panel controls is not more biased than the recruitment of controls via RDD; however, it cannot be excluded that both methods produced results that were biased similarly.

The difference in education level did not affect the main results of the CCS. Both control groups reported similar frequencies of fruit consumption, including melons, which were identified as the infection vehicle. Systematic differences between the distribution of demographic characteristics between the panel and the general population have been addressed and discussed in the previous publication of the group [14] and were addressed by applying frequency matching (on age and sex) to ensure that the data collected on controls better reflected the population at risk.

Since recruiting panel controls is faster than classical control recruitment, the time lag between interview and queried exposure time period of panel controls is more comparable to the time lag between interview and exposure time period before disease onset. Long delays between exposure period and interviews may result in a recall bias. Indeed, healthy controls are less likely to remember in more detail what they ate than cases, who may easily attempt to figure out what food items could have made them sick [20]. Panel control memory was easily assisted by including the pictures of food items and melon types in the online questionnaire when classical controls received a verbal description of melon types on the telephone.

The consumption of melons was 2–3 times more strongly associated with the disease when controls were asked about their fruit consumption in April (when the outbreak occurred) than in July (when they were interviewed). This applies to both panel and classical controls. A plausible explanation for that is the seasonality of melon consumption, as these fruits are more likely to be eaten during summer months, including July. To consider solely the melon consumption in July would have resulted in an underestimation of the association between melon consumption and disease. To avoid this bias, we queried controls about their melon consumption in April, the period covering the time of the outbreak.

In both models including panel controls (A and C), two other fruit items (orange and kiwi) were positively associated with the disease, albeit not as strongly as melons. A possible explanation is that controls and/or cases did not recall the consumption of these fruits accurately, or consumers of melons presumably also often consume other fruits. There was no other evidence from the multinational outbreak investigation that pointed towards oranges or kiwis as possible infection vehicles, whereas the microbiological investigation identified the outbreak sequence type in Galia melons from Honduras, confirming the analytical-epidemiological evidence from the CCS in the UK and Germany [17].

The value of analytical studies is sometimes discussed in opposition to microbiological evidence from discriminatory methods like whole-genome sequencing (WGS). This point of view overlooks inherent limitations of the WGS such as missing information on different exposures (e.g. food items) of cases or the difficulty to obtain microbiological evidence for short-life products like salad, vegetables, and fruits [21]. In the present outbreak, the epidemiological evidence suggesting melons as a probable vehicle of infection became known before the microbiological findings were available [15]. These points underline the advantage of combining analytical epidemiology and WGS in foodborne outbreak investigations.

One limitation of the study lies in the low number of panel controls in the younger age categories, which impaired a complete frequency matching according to the intended case–control ratio. The second limitation derives from the lack of representativeness of the HuGO panel for the German population as the panel only includes participants from the federal state of Lower Saxony. Both limitations may have resulted in a smaller study power to detect the infection vehicle. This was not relevant in the present study because the association between being a case and consumption of Galia melons – the infection vehicle – was very high. However, this could possibly negatively influence the ability of a CSS to identify a contaminated food item when investigating outbreaks where multiple infection vehicles with weaker associations may be suspected, or where the spatial distribution of exposure is inhomogeneous.

## Conclusion

The study provides valuable evidence for the proof of concept of using an online panel as a source of controls in a CCS design in order to investigate a foodborne outbreak. During an ongoing foodborne outbreak, two control groups (classical and panel controls) were deployed in a CCS in parallel. With a systematic methodology to assess their feasibility, suitability, and reliability, both methods were comprehensively compared from their implementation to the analysis of results.

Comparing panel to classical controls, the results did not differ in successfully confirming the most likely infection vehicle, Galia melons, in a foodborne *S.* Braenderup outbreak in 2021. Using panel controls was more efficient regarding time, costs, and human resources than recruiting and interviewing controls by telephone.

Particularly the more timely identification of the infection vehicle in foodborne outbreaks could provide an important public health benefit.

We recommend to foster research to identify in which circumstances panel members can be used as controls in CCS designs. This is of particular public health relevance as this low-threshold and efficient method could become a valuable option in outbreak investigations.

## Data Availability

Data supporting the findings of the study are openly available in Zenodo at https://doi.org/10.5281/zenodo.7333295, reference number 7333295.

## Supplementary material

For supplementary material accompanying this paper visit http://doi.org/10.1017/S0950268823000493. click here to view supplementary material
