# Supplementary material for: Recruiting controls from an online panel for a case–control study enabled a timely and reliable foodborne Salmonella outbreak investigation, Germany 2021
Source: Epidemiol Infect. 2023 Apr 5;151:e70. doi: 10.1017/S0950268823000493 (PMC10204133; doi:10.1017/S0950268823000493)
Supplement: Supplementary file 1 [file S0950268823000493sup001.docx]

**Supplementary Material**

Epidemiology and Infection “Recruiting controls from an online panel for a case-control study enabled a timely and reliable foodborne Salmonella outbreak investigation, Germany 2021”

**Author’s names**

Anna Luczynska ^1,2^,* Johannes Dreesman^2^, Elke Mertens ^2^, Mareike Wollenweber^2^, Delphine Perriat^3**^, Bettina Rosner^3**^

*Corresponding author

**Contributed equally to this work.

**Affiliations:**

^1^ ECDC Fellowship Programme, Field Epidemiology path (EPIET), European Centre for Disease Control and Prevention (ECDC), Stockholm, Sweden

^2^ Public Health Agency of Lower Saxony, Department for Infectious Disease Epidemiology, Germany;

^3^ Department of Infectious Disease Epidemiology, Robert Koch Institute, Berlin, Germany

**Supplementary Table S1.** Frequency-matching on age group and sex between cases and panel controls, case-control study to investigate the *S.* Braenderup outbreak, Germany 2021.

|  | **female** | | | **male** | | |
| --- | --- | --- | --- | --- | --- | --- |
| **age group (years)** | **cases** | **panel controls** | **frequency-matched panel controls** | **cases** | **panel controls** | **frequency-matched panel controls** |
| 1-17 | 4 | 12 | 12 | 5 | 13 | 13 |
| 18-35 | 7 | 10 | 10 | 2 | 4 | 4 |
| 36-59 | 4 | 41 | 12 | 0 | 20 | 0 |
| ≥60 | 4 | 28 | 12 | 6 | 27 | 18 |
| **total** | 19 | 91 | 46 | 13 | 64 | 35 |

Panel controls were randomly selected to reach a case:control ratio of 1:3 in each age-sex category. When the intended case-control ratio was not feasible to obtain, lower control number in that category were accepted: two controls are missing among men in the age category 1-17 years, 2 controls are missing among men in the age category 18-35, and 11 controls are missing among women in the age category 18-35 years

**Supplementary Table S2**. Food exposures among classical controls (reference) and panel controls, case-control study to investigate the *S.* Braenderup outbreak, Germany, 2021.

|  | FM classical controls^a^  exposed 7 days after Easter Sunday (05-11 April 2021)  n (%)  (n_tot_=110) | FM panel controls^b^  exposed 7 days after Easter Sunday (05-11 April 2021)  n (%)  (n_tot_=81) | FM classical controls vs exposed FM panel controls | |
| --- | --- | --- | --- | --- |
|  |  |  | **aOR (95% CI)** | ***p*-value (Fisher)** |
| Apple | 91 (84) | 60 (74) | 0.53 [0.24-1.2] | 0.10 |
| Blueberry | 44 (40) | 22 (27) | 0.56 [0.28-1.1] | 0.09 |
| Blackberry | 3 (2.7) | 3 (3.7) | 1.4 [0.18-11] | 0.70 |
| Raspberry | 26 (24) | 18 (22) | 0.92 [0.44-1.9] | 0.86 |
| Strawberry | 40 (36) | 23 (28) | 0.69 [0.35-1.4] | 0.28 |

^a^ FM classical controls: frequency-matched classical controls i.e. adults recruited through a classical method (random digit dialling), that are frequency-matched to adult cases on age group, sex, and federal state of residency; or children recruited through a classical method (random digit dialling) that are frequency-matched to children cases by age group and federal state of residency.

^b^FM panel controls: frequency-matched panel controls i.e. adult panel members that are frequency-matched to adult cases on sex and age group; or children of adult panel ^c^Galia or Cantaloupe or Honeydew melon or similar type of melon (composite variable)

aOR: odds ratio adjusted for age and sex; CI: confidence interval; n_tot_: total number of participants in the study group

**Supplementary Table S3**. Food exposures among classical controls (reference) and panel controls, case-control study to investigate the *S.* Braenderup outbreak, Germany, 2021.

|  | FM classical controls^a^  exposed 7 days before the interview (July 2021)  n (%)  (n_tot_=110) | FM panel controls^b^  exposed 7 days before the interview (July 2021)  n (%)  (n_tot_=81) | FM classical controls vs FM panel controls | |
| --- | --- | --- | --- | --- |
|  |  |  | **OR (95% CI)** | ***p*-value (Fisher exact test)** |
| **Melons** |  | | | |
| Any melon^c^ | 17 (15) | 11 (14) | 0.86 [0.34-2.1] | 0.84 |
| Galia melon | 3 (2.7) | 6 (7.4) | 2.9 [0.58-18] | 0.17 |
| Cantaloupe melon | 7 (6.3) | 5 (6.2) | 0.97 [0.23-3.7] | 1.0 |
| Honeydew melon | 11 (10) | 7 (8.6) | 0.85 [0.27-2.5] | 0.81 |
| Water melon | 51 (46) | 48 (59) | 1.7 [0.91-3.1] | 0.08 |
| Others | 1 (0.91) | 1 (1.2) | 1.4 [0.02-108] | 1.0 |
| **Other fruit items** |  |  |  |  |
| Cherry | 60 (55) | 31 (38) | 0.52 [0.28-0.96] | 0.029* |
| Mango | 23 (21) | 8 (9.9) | 0.41 (0.15-1.04) | 0.048* |
| Orange | 9 (8.2) | 11 (14) | 1.8 [0.63-5.1] | 0.24 |
| Pineapple | 14 (13) | 17 (21) | 1.8 (0.78-4.3] | 0.16 |
| Pear | 21 (19) | 18 (22) | 1.2 [0.56-2.6] | 0.59 |
| Grape | 44 (40) | 39 (48) | 1.4 [0.75-2.6] | 0.30 |
| Apple | 72 (65) | 56 (69) | 1.2 [0.61-2.3] | 0.64 |
| Blueberry | 36 (33) | 25 (31) | 0.92 [0.47-1.8] | 0.88 |
| Banana | 62 (56) | 43 (53) | 0.88 [0.47-1.6] | 0.66 |
| Nectarine | 44 (40) | 32 (40) | 0.98 [0.52-1.8] | 1.00 |
| Peach | 33 (30) | 23 (28) | 0.93 [0.47-1.8] | 0.87 |
| Strawberry | 96 (87) | 70 (86) | 0.93 [0.37-2.4] | 1.00 |
| Lime | 17 (15) | 5 (6.2) | 0.36 [0.10-1.1] | 0.065 |
| Lemon | 50 (45) | 36 (44) | 0.96 [0.52-1.8] | 1.00 |
| Plum | 7 (6.4) | 7 (8.6) | 1.4 [0.39-4.8] | 0.59 |
| Kiwi | 20 (18) | 11 (14) | 0.71 [0.29-1.7] | 0.43 |
| Raspberry | 52 (47) | 34 (42) | 0.81 [0.43-1.5] | 0.56 |
| Other type of berry | 37 (34) | 22 (27) | 0.74 [0.37-1.4] | 0.35 |
| Apricot | 44 (40) | 23 (28) | 0.59 [0.31-1.2] | 0.12 |
| Tangerine | 6 (5.5) | 2 (2.5) | 0.44 [0.04-2.6] | 0.47 |
| Blackberry | 10 (9.09) | 6 (7.4) | 0.80 [0.23-2.6] | 0.80 |
| Pomegranate | 5 (4.6) | 2 (2.5) | 0.53 [0.05-3.4] | 0.70 |
| Papaya | 1 (0.91) | 0 (0) | 0.00[0.00-53] | 1.00 |

^a^ FM classical controls: frequency-matched classical controls i.e. adults recruited through a classical method (random digit dialling), that are frequency-matched to adult cases on age group, sex, and federal state of residency; or children recruited through a classical method (random digit dialling) that are frequency-matched to children cases by age group and federal state of residency.

^b^FM panel controls: frequency-matched panel controls i.e. adult panel members that are frequency-matched to adult cases on sex and age group; or children of adult panel OR: odds ratio; 95% CI: 95% confidence interval; n_tot_: total number of participants in the study group

^c^Galia or Cantaloupe or Honeydew melon or similar type of melon (composite variable)

* significant association between the fruit consumption and the disease, with p-value of Fisher exact test < 0.05

OR: odds ratio; CI: confidence interval; n_tot_: total number of participants in the study group

**Supplementary Table S4.** Single-variable logistic regression analyses (adjusted for age group and sex), case-control study to investigate the *S*.Braenderup outbreak, Germany, 2021.

|  | **Cases**  **exposed 7 days before disease onset**  **(n_tot_=32)** | **FM classical controls^a^**  **exposed 7 days after Easter Sunday (05-11 April 2021)**  **(n_tot_=110)** | | | **FM panel controls^b^**  **exposed 7 days after Easter Sunday (05-11 April 2021)**  **(n_tot_=81)** | | |
| --- | --- | --- | --- | --- | --- | --- | --- |
|  | **n (%)** | **n (%)** | **aOR^b^ [95% CI**] | ***p*-value** | **n (%)** | **aOR^b^ [95% CI**] | ***p*-value** |
| **Melons** |  |  |  |  |  |  |  |
| Any melon^c^ | 19 (59) | 6 (5.5) | 32 [11-120] | <0.001 | 7 (8.6) | 19 [6.3-71] | <0.001 |
| Galia melon | 12 (38) | 1 (0.91) | 96 [15-2100] | <0.001 | 3 (3.7) | 18 [4.6-100] | <0.001 |
| Cantaloupe melon | 9 (28) | 2 (1.8) | 29 [6.2-230] | <0.001 | 1 (1.2) | 27 [4.4-540] | 0.003 |
| Honeydew melon | 12 (38) | 6 (5.5) | 10 [3.6-34] | <0.001 | 5 (6.2) | 8.6 [2.7-31] | <0.001 |
| Water melon | 10 (31) | 13 (12) | 3.7 [1.4-10] | 0.010 | 9 (11) | 5.0 [1.6-16] | 0.006 |
| Others | 2 (6.3) | 0 (0.00) | n.d. |  | 0 (0.00) | n.d. |  |
| **Other fruit items** |  |  |  |  |  |  |  |
| Apple | 29 (91) | 91 (84) | 2.0 [0.58-9.5] | 0.31 | 60 (74) | 3.4 [1.0-16] | 0.069 |
| Blueberry | 13 (42) | 44 (40) | 1.1 [0.45-2.4] | 0.90 | 22 (27) | 2.1 [0.85-5.4] | 0.11 |
| Raspberry | 4 (13) | 26 (24) | 0.42 [0.11-1.2] | 0.15 | 18 (22) | 0.59 [0.16-1.8] | 0.38 |
| Strawberry | 11 (35) | 40 (36) | 0.91 [0.38-2.1] | 0.83 | 23 (28) | 1.4 [0.54-3.4] | 0.50 |
| Blackberry | 0 (0.0) | 3 (2.7) | n.d. |  | 3 (3.7) | n.d. |  |

^a^ FM classical controls: frequency-matched classical controls i.e. adults recruited through a classical method (random digit dialling), that are frequency-matched to adult cases on age group, sex, and federal state of residency; or children recruited through a classical method (random digit dialling) that are frequency-matched to children cases by age group and federal state of residency.

^b^FM panel controls: frequency-matched panel controls i.e. adult panel members that are frequency-matched to adult cases on sex and age group; or children of adult panel ^c^Galia or Cantaloupe or Honeydew melon or similar type of melon [composite variable)

aOR: odds ratio adjusted for age and sex; 95% CI: 95% confidence interval; n_tot_: total number of participants in the study group; n.d.: not determined

**Supplementary Table S5.** Single-variable logistic regression analyses (adjusted for age group and sex), case-control study to investigate the *S*. Braenderup outbreak, Germany, 2021.

|  | **Cases**  **exposed 7 days before disease onset**  **(n_tot_=32)** | **FM classical controls^a^**  **Exposed 7 days before the interview (July 2021)**  **(n_tot_=110)** | | | **FM panel controls^a^**  **Exposed 7 days before the interview (July 2021)**  **(n_tot_=81)** | | |
| --- | --- | --- | --- | --- | --- | --- | --- |
|  | **n (%)** | **Exposed, n (%)** | **aOR^b^ (95% CI**] | ***p*-value** | **n (%)** | **aOR (95% CI**] | ***p*-value** |
| **Melons** |  |  |  |  |  |  |  |
| Any melon^c^ | 19 (59) | 17 (15) | 10 [4.1-28] | <0.001 | 11 (14) | 11 [4.0-34] | <0.001 |
| Galia melon | 12 (38) | 3 (2.7) | 33 [8.4-180] | <0.001 | 6 (7.4) | 9.07 [2.8-34] | <0.001 |
| Cantaloupe melon | 9 (28) | 7 (6.4) | 7.0 [2.2-23] | 0.001 | 5 (6.2) | 6.5 [1.8-28] | 0.007 |
| Honeydew melon | 12 (38) | 11 (10) | 5.8 [2.2-16] | <0.001 | 7 (8.6) | 5.8 [2.0-19] | <0.001 |
| Water melon | 10 (31) | 51 (46) | 0.47 [0.18-1.1] | 0.096 | 48 (59) | 0.24 [0.08-0.64] | 0.006 |
| Others | 2 (6.3) | 1 (0.9) | 11 [0.82-300] | 0.084 | 1 (1.2) | 3.3 [0.12-90] | 0.41 |
| **Other fruit items** |  |  |  |  |  |  |  |
| Orange | 12 (39) | 9 (8.2) | 7.8 [2.9-23] | <0.001 | 11 (14) | 5.1 [1.9-15] | 0.002 |
| Apple | 29 (91) | 72 (65) | 5.7 [1.8-26] | 0.008 | 56 (69) | 4.4 [1.4-20] | 0.026 |
| Tangerine | 5 (16) | 6 (5.5) | 4.3 [1.0-19] | 0.047 | 2 (2.5) | 11 [2.0-100] | 0.010 |
| Pineapple | 11 (35) | 14 (13) | 3.8 [1.4-9.9] | 0.006 | 17 (21) | 2.1 [0.79-5.5] | 0.14 |
| Kiwi | 11 (37) | 20 (18) | 2.7 [1.1-6.4] | 0.032 | 11 (14) | 4.3 [1.5-13] | 0.008 |
| Banana | 24 (75) | 62 (56) | 2.6 [1.1-6.7] | 0.045 | 43 (53) | 3.3 [1.3-9.5] | 0.017 |
| Papaya | 1 (3.2) | 1 (0.91) | 5.2 [0.18-150] | 0.27 | 0 (0.00) | n.d. |  |
| Pear | 9 (30) | 21 (19) | 1.9 [0.71-4.7] | 0.19 | 18 (22) | 1.8 [0.66-4.9] | 0.24 |
| Grape | 16 (52) | 44 (40) | 1.5 [0.67-3.6] | 0.30 | 39 (48) | 1.3 [0.54-3.3] | 0.55 |
| Pomegranate | 2 (6.5) | 5 (4.6) | 1.5 [0.20-7.4] | 0.66 | 2 (2.5) | 2.5 [0.27-24] | 0.39 |
| Blueberry | 13 (42) | 36 (33) | 1.5 [0.62-3.4] | 0.38 | 25 (31) | 2.0 [0.78-5.0] | 0.15 |
| Plum | 3 (9.7) | 7 (6.4) | 1.5 [0.3-5.8] | 0.62 | 7 (8.6) | 1.1 [0.22-4.4] | 0.91 |
| Mango | 8 (26) | 23 (21) | 1.3 [0.48-3.4] | 0.58 | 8 (9.9) | 3.4 [1.08-11] | 0.035 |
| Lemon | 13 (42) | 50 (45) | 0.90 [0.39-2.0] | 0.80 | 36 (44) | 1.01 [0.41-2.4] | 0.99 |
| Lime | 2 (6.5) | 17 (15) | 0.35 [0.05-1.4] | 0.19 | 5 (6.2) | 1.2 [0.16-6.4] | 0.81 |
| Nectarine | 6(19) | 44(40) | 0.35 [0.12-0.88] | 0.035 | 32 (40) | 0.36 [0.12-0.95] | 0.051 |
| Raspberry | 4 (13) | 5 (47) | 0.16 [0.05-0.46] | 0.002 | 34 (42) | 0.19 [0.05-0.57] | 0.006 |
| Peach | 2 (6.4) | 33 (30) | 0.15 [0.02-0.57] | 0.016 | 23 (28) | 0.17 [0.02-056] | 0.023 |
| Apricot | 2 (6.5) | 44 (40) | 0.10 [0.02-0.37 | 0.003 | 23 (28) | 0.18 [0.03-0.68] | 0.027 |
| Strawberry | 11 (35) | 96 (87) | 0.08 [0.03-0.19] | <0.001 | 70 (86) | 0.09 [0.03-0.23] | <0.001 |
| Cherry | 1 (3.2) | 60 (55) | 0.03 [0.0-0.13] | <0.001 | 31 (38) | 0.05 [0.02-0.27] | 0.005 |
| Blackberry | 0 (0.0) | 0 (0.0) | n.d. |  | 6 (7.4) | n.d. |  |
| Other type of berry | 0 (0.0) | 0 (0.0) | n.d. |  | 22 (27) | n.d. |  |

^a^ FM classical controls: frequency-matched classical controls i.e. adults recruited through a classical method (random digit dialling), that are frequency-matched to adult cases on age group, sex, and federal state of residency; or children recruited through a classical method (random digit dialling) that are frequency-matched to children cases by age group and federal state of residency.

^b^FM panel controls: frequency-matched panel controls i.e. adult panel members that are frequency-matched to adult cases on sex and age group; or children of adult panel

^c^Galia or Cantaloupe or Honeydew melon or similar type of melon [composite variable)

aOR: odds ratio adjusted for age and sex; 95% CI: 95% confidence interval; n_tot_: total number of participants in the study group; n.d.: not determined
